# Assessment of unmet needs to address noncompliant households during polio supplemental immunization activities in Kaduna state, 2014–2016

**DOI:** 10.1186/s12889-018-6192-0

**Published:** 2018-12-13

**Authors:** Hadiza Aliyu Iyal, Faisal Shuaib, Madubu Dauda, Abdullahi Suleiman, Fiona Braka, Sisay G. Tegegne, Peter Nsubuga, Terna Nomhwange, Yared G. Yehualashet, Sambo Ishaku, Charity Warigon, Furera Zakari, Gregory Umeh, Lami Samaila, Basirat Abdullahi, Kulchumi Hammanyero, Paul Dogo, Dawud Adamu, Rui G. Vaz, Wondimagegnehu Alemu

**Affiliations:** 1 World Health Organization Country Office, Abuja, Nigeria; 2 National Primary Health Care Development Agency, Abuja, Nigeria; 3 Global Public Health Solutions, Atlanta, GA USA; 4 State Ministry of Health, Kaduna, Nigeria

**Keywords:** Unmet needs, Noncompliant households, Supplementary immunization activities, Unmet need intervention, High-risk communities, Vaccine rejection

## Abstract

**Background:**

Despite concerted global efforts being made to eradicate poliomyelitis, the wild poliovirus still circulates in three countries, including Nigeria. In addition, Nigeria experiences occasional outbreaks of the circulating vaccine-derived poliovirus type 2 (cVDPV2). Vaccine rejection by caregivers persists in some parts of northern Nigeria, which compromises the quality of supplemental immunization activities (SIAs). In 2013, the Expert Review Committee (ERC) on polio recommended innovative interventions in all high-risk northern states to improve the quality of SIA rounds through innovative interventions. The study assessed the impact of using unmet needs data to develop effective strategies to address noncompliant households in 13 high-risk Local government areas (LGAs) in Kaduna state, Nigeria.

**Methods:**

A retrospective study was conducted in noncompliant communities using unmet needs data collated from 2014 to 2016. Household-based noncompliance data collated from tally sheets between 2013 and 2016 was also analyzed to assess the impact of unmet needs data in addressing noncompliance households in high-risk communities in Kaduna state. A structured interview was used to interview caregivers by the application of an unmet needs questionnaire, a quantitative study that assesses caregiver perception on immunization and other unmet needs which, if the gaps were addressed, would allow them to accept immunization services. Interventions include siting of temporary health camps in noncompliant communities to provide free medical consultations, treatment of minor ailments, provision of free antimalaria drugs and other essential drugs, and also referral of serious cases; intervention of religious and traditional leaders, youth against polio intervention, and the use of attractive bonuses (sweets, balloons, milk) during SIAs were all innovations applied to reduce noncompliance in households in affected communities as the need for eradication of polio was declared as a state of emergency. Outcomes from the analyses of unmet needs data were used to direct specific interventions to certain areas where they will be more effective in reducing the number of noncompliant households recorded on the tally sheet in each SIA round. Hence, seven immunization parameters were assessed from the unmet needs data.

**Results:**

Overall, 54% of the noncompliant caregivers interviewed were ready to support immunization services in their communities. The majority of caregivers were also willing to vaccinate their children publicly following unmet needs interventions that were conducted in noncompliant communities. The trend of noncompliant households decreased by 79% from 16,331 in September 2013 to 3394 in May 2016.

**Conclusions:**

Unmet needs interventions were effective in reducing the number of noncompliant households recorded during SIA rounds in Kaduna State. Hence, unmet needs intervention could be adapted at all levels to address challenges faced in other primary healthcare programs in Nigeria.

## Background

Poliomyelitis is a debilitating disease that causes permanent disability in humans and has been targeted for eradication through strategies of the global polio eradication initiative (GPEI) [1, 2]. Supplemental immunization activities (SIAs) are one of the components of the GPEI strategy [3]. The oral polio vaccine (OPV) is an effective vaccine against poliomyelitis infection and has interrupted polio transmission in many regions [4, 5]. Nigeria has used innovative interventions to reduce polio transmission in most parts of northern Nigeria [6–8]. However, OPV acceptance has been a major challenge in the implementation of SIAs, especially in the high-risk northern states due to misconceptions about the vaccine [1, 9, 10].

Since the declaration of poliomyelitis as an emergency in Nigeria in 2012, an Expert Review Committee (ERC) was set up for the polio emergency program the following year. The ERC recommended innovative interventions to improve the quality of SIA rounds and overcome implementation challenges in all high-risk northern states in Nigeria [11–13]. Kaduna state remains at high risk due to persistent pockets of noncompliant households and missed children during monitoring exercises that follow SIAs. These serve as a major threat to the GPEI program in Kaduna state and, as such, different innovative activities were implemented during SIAs using the unmet needs tool as a guide.

The unmet needs tool is a questionnaire designed by the World Health Organization (WHO) country office in Nigeria. The authors conducted content validity of the study tool and were convinced that the variables developed accurately assessed what needed to be measured. The tool is a primary instrument that guides the channelling of innovative interventions to high-risk communities in Kaduna state, based on unmet needs. The unmet needs interventions in Kaduna are mainly focused on 13 high-risk Local Government Areas (LGAs) for poliomyelitis disease. The questionnaire assesses knowledge gaps for caregivers on the immunization process, and the persons whom they would trust to convince them to provide full immunization for their children. It also assesses the preferred credible source of information for caregivers on immunization and suggests additional interventions alongside immunization services. Other things assessed were the readiness of caregivers to support immunization in their communities and ways to provide such support in their immediate environment. The unmet needs tool provides insight on how to mitigate the wrong perceptions caregivers have towards immunization. The tool also enables determination of the extent of resolution or persistence of noncompliant households.

Administration of the unmet needs tool has led to improved SIA coverage and access to highly susceptible eligible children. The susceptible children are now reached during SIA rounds as a result of channeling the right intervention to the appropriate communities to address their unmet needs. Furthermore, the findings give insight into other pressing issues aside from polio vaccination that can be tackled to improve the uptake of other healthcare interventions.

This study describes how the unmet needs data tool was applied in each round, from 2014 to 2016, to gain insight into the reasons for the caregiver to be noncompliant during the SIA rounds. We also describe how the data were used to develop strategies and assessed whether using the tool had an effect on noncompliant households in Kaduna state.

## Methods

We analyzed two datasets: the unmet needs data collated from noncompliant communities from 2014 to 2016 in the 21 rounds of SIAs conducted in Kaduna state, and noncompliance data recorded in the state from 2013 to 2016.

### Ethical approval

Ethical approval was obtained from the Kaduna state Ministry of Health ethical review committee to carry out this study.

### Study design

This was a retrospective analysis of unmet needs using data from 2014 to 2016 in noncompliant communities in Kaduna state.

### Study site

Kaduna state was identified among the10 high-risk northern states of Nigeria. It was ranked third in the country, accounting for 14% of the total wild poliovirus (WPV) burden in Nigeria in 2012. The last WPV case recorded in the state was in November 2012. All the WPV cases recorded were from the northern LGAs of the state [14]. More attention was given to 13 identified northern LGAs compared with the southern part of the state because of the high risk of transmission of poliovirus and rejection of OPV. The unmet needs tool was consistently administered to noncompliant households identified in these 13 northern LGAs.

### Study population

The study targeted caregivers with children under 5 years of age from noncompliant households in 13 high-risk LGAs in Kaduna state. The majority of the caregivers were from a low socioeconomic class, with poor literacy levels, and lacking basic amenities such as potable water, electricity, and infrastructure, and with limited access to affordable medical care.

### Procedures

Field volunteers (FVs) were trained by WHO staff on how to apply the unmet needs tool designed by WHO in noncompliant communities. A total of 50 households from five settlements in each of the 13 high-risk LGAs were considered for the unmet needs intervention during each SIA round. Ten households per noncompliant community in the five selected settlements were randomly visited during each round. The unmet needs questionnaire was administered to parents or caregivers of children under 5 years of age after they consented. Questionnaires were always collated after each round to direct specific innovations to different noncompliant communities to increase the uptake of OPV in next coming SIA round in Kaduna state. Overall, a total of 12,120 households were visited in 129 of the 255 political wards of the state (> 50%) between 2014 and 2016.

Additionally, tally sheet data were collated after each SIA round to compile the total number of noncompliant households and children involved for each LGA by settlement. Tally sheets are the primary source for data used by the SIA team to capture information on immunization activities including noncompliant households and children involved in each SIA round. The data on the noncompliant households were collated, and communities where these households reside were listed. FVs were assigned to visit these communities at each SIA round and to randomly select from the noncompliant households and administer the unmet needs questionnaire. Our study analyzed the tally sheet noncompliant data component recorded at each SIA rounds from 2013 to 2016. The trend of noncompliant households following the introduction of innovative interventions to address unmet needs was observed.

### Data management

Unmet needs questionnaires collated over a period of 3 years (from 2014 to 2016) for the 21 rounds of SIAs conducted in Kaduna state were analyzed. A total of 12,120 questionnaires out of the expected 13,650 generated from the field were analyzed using Microsoft Excel. The data were cleaned, and 504 questionnaires were discarded due to data quality issues or incomplete or zero data from some of the selected LGAs and failure to use drop-down buttons provided on the template to choose from the options provided. Hence, for these reasons, the unmet needs data were not considered from 2013 when the intervention was first introduced in the state.

We also analyzed the trend of identified noncompliant households recorded from the state primary data (tally sheets) from 2013 to 2016.

## Results

Respondents interviewed were from 1405 noncompliant settlements in 13 high-risk LGAs of Kaduna state in 2014–2016 (Table 1).

**Table 1:** Distribution of unmet needs questionnaires administered in Kaduna state, 2014–2016

Year	Local Government Area	Wards	Settlements	Households
2014	13	137	462	4774
2015	13	147	588	5257
2016	13	104	355	2089
Total	–	–	1405	12,120

The immunization knowledge gaps of caregivers showed that 3107 (27%) wanted to know why so many SIA rounds are conducted in Kaduna state. A total of 2508 (22%) and 831 (7%) of respondents were concerned about OPV safety and the benefits of OPV, and want the program implementers to shed more light on these. A total of 2318 (20%) caregivers want to know why only OPV is given on a regular basis before they will allow their children to get vaccinated. Also, among the respondents, 1051(9%) and 226 (2%) were worried about possible side effects and risk of paralysis associated with regular use of OPV. Finally, a total of 1575 (14%) of the respondents had gaps that fell under none of the above options in the period 2014–2016 (Table 2).

**Table 2:** Proportion of immunization knowledge gaps questioned/requested by caregivers in Kaduna state, 2014–2016

	2014	2015	2016	Average
Benefits of oral polio vaccine (OPV)	7%	8%	6%	7%
OPV safety	21%	22%	22%	22%
Risk of paralysis	1%	2%	3%	2%
Side effects of OPV	7%	10%	11%	9%
Why so many rounds	27%	25%	28%	27%
Why only OPV	20%	20%	19%	20%
None of the above/others	17%	13%	11%	14%

A total of 3290 (28%) caregivers prefer to be mobilized by religious leaders for vaccination, 2633 (23%) by qualified health workers, and 1781 (15%) and 1316(11%) by traditional leaders and community leaders, respectively. We also found that political leaders, relatives, or others have roles in mobilizing 697 (6%) noncompliant caregivers each, while mobilization percentages through mass media and town announcers were 2% each (Table 3).

**Table 3:** Proportion of suggested different groups trusted by respondents from our study to convince for full immunization

	2014	2015	2016	Average
Traditional leader	19%	12%	15%	15%
Religious leader	31%	22%	32%	28%
Qualified health worker	25%	24%	19%	23%
Mass media (radio/television)	1%	3%	3%	2%
Town announcer	1%	2%	3%	2%
Community leader	12%	13%	9%	11%
Relative	5%	7%	6%	6%
Political leader	5%	8%	5%	6%
Others	1%	9%	8%	6%

The suggestions made by caregivers for added interventions alongside immunization which would have made them fully immunize their children were free medical consultations in 2904 (25%) and discounted drugs in 2284 (20%). Others (e.g., electricity, food, and fertilizer) were mentioned by 1588 (14%) and antimalarials were mentioned by 1355 (12%) caregivers. Insecticide-treated nets (ITNs), hospital care, and antenatal care (ANC) accounted for 891 (8%) each. The majority of caregivers felt that polio is not a priority because these basic needs have not been met by the government. Respondents who requested other routine immunization antigens constituted 736 (6%), which represents the least among all parameters in 2014–2016 (Table 4).

**Table 4:** Proportion of additional interventions alongside immunization requested by caregivers in Kaduna state, 2014–2016

	2014	2015	2016	Average
Antimalarial	10%	9%	16%	12%
Antenatal clinic	5%	8%	10%	8%
Free medical consultations	42%	18%	15%	25%
Hospital/clinic	6%	8%	9%	8%
Insecticide-treated nets	10%	9%	6%	8%
Other antigens	8%	5%	6%	6%
Free/discounted drugs	7%	30%	22%	20%
Others	12%	13%	16%	14%

Overall, the majority of the parents (6304 (54%) of 11,616) responded positively to supporting immunization services in their communities; 3309 (29%) were not ready to support immunization activities, and 2004 (17%) gave no response. Out of the 6304 respondents who were ready to support immunization in their communities, 3379 (54%) were ready to vaccinate their children publicly. A total of 1525 (24%) were prepared to sensitize other noncompliant families. Furthermore, 1399 (22%) respondents were ready to convince neighbors, family members, and others to accept vaccination for their children.

**Fig. 1:**
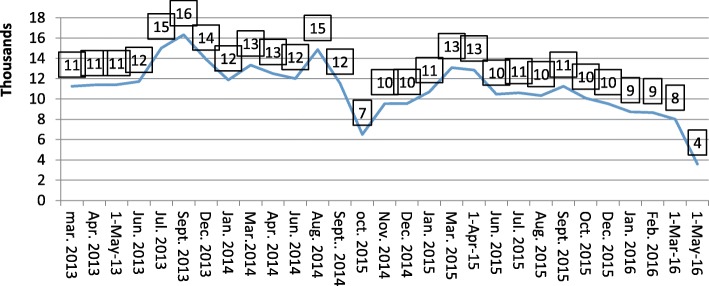
Trend of identified noncompliant households documented during SIAs in Kaduna state, 2013–2016

The trend of identified noncompliant households documented during SIAs in Kaduna state from 2014 to 2016 showed a steady decline in the trend, especially in the data observed in 2015–2016. The number of noncompliant households reduced over time by 79% from 16,331 in September 2013 to 3394 as in May 2016 (Fig. 1).

## Discussion

The unmet needs tool was successful because the data generated helped identify and address problems peculiar to noncompliant communities and interventions such as sensitization and community dialogue meetings with noncompliant caregivers on the benefits of OPV and the reasons for “too many rounds” of OPV campaigns. Also, provision of free medical consultations and free discounted drugs, the engagement of religious leaders, traditional leaders, youth, and edutainment programs were directed to noncompliant communities based on the identified unmet needs of the caregivers. There was an increase in OPV uptake among caregivers and a reduction in the number of noncompliant households recorded in Kaduna state as a result of fulfilling their unmet needs. The unmet needs intervention was effective in changing the negative attitude of some parents on polio immunization programs, with a majority of 54% willing to support immunization in their communities in our study.

We found respondents from noncompliant communities during the period under review had a large knowledge gap on the polio immunization program in Kaduna state. A knowledge gap on immunization could be one of the major contributing factors to vaccine rejection for their children. Most caregivers interviewed wanted to know from program implementers why there are too many rounds of OPV campaigns (27%) and why there is the use of only OPV (22%) for a disease they rarely see and not antigens against malaria or measles instead that affects their children more often than polio. There were also some respondents who demanded to be educated on OPV safety (22%), its benefits (7%), the side effects of OPV (22%), and the risk of paralysis that could be associated with frequent use of the vaccine. The findings were in line with a similar study on assessment of reasons for OPV refusal in five northern states of Nigeria conducted in 2012 by Michael et al. [15] where the results showed that 59% of respondents had knowledge gap on polio immunization.

The complaint of too many rounds of OPV campaigns continued to affect the program since the number of rounds did not decrease over time; this made it difficult for parents to comprehend and, subsequently, raised suspicion on the use of OPV and resulted in low uptake among caregivers, especially in the northern region of Nigeria [16]. Noncompliant communities incited their youths to threaten vaccination team members not to visit their communities again to immunize children. An innovative approach was applied to engage youths from these communities as part of the vaccination teams which helped to overcome threats to vaccination teams and address noncompliance [8].

Mobilization is one vital component of immunization that encourages caregivers to turn up for immunization services [17]. We found out that most parents from the noncompliant communities have more faith in religious leaders and trust this group more than traditional leaders or qualified health workers to convince them to accept full immunization for their children. WHO Kaduna state supports the engagement of religious leaders, Mallams, Quranic teachers, health workers, and traditional leaders to convince parents on the importance of immunization. These groups are deployed to specific areas where such concerns were raised to address the unmet needs of the target group living in those areas on the need to willingly allow immunization for their children.

Furthermore, in response to the suggested added interventions that were preferred by caregivers, many of the caregivers requested free medical consultation and drugs. Thus, temporary health camps manned by health workers that provide free consultations and free or discounted drugs are provided to such communities to meet their unmet needs. Some respondents wanted to have insecticide-treated mosquito nets and antenatal care services provided alongside immunization services, whereas others requested power supply, portable water, food, good roads, and fertilizer among other things from the government. Similar findings were mentioned in the study conducted in northern Nigeria in 2014 on the reasons for refusal of OPV [15], where 33% of the respondents urged the government to address other unmet health and social needs of the populace.

Surprisingly, there were very few caregivers who requested other routine immunization (RI) antigens. Hence, there was a low demand for routine immunization (probably due to lack of adequate understanding of the importance of RI antigens) as was also observed by Wonodi et al. [18] among some of the respondents interviewed from eight states in Nigeria in 2012. The value of RI was underestimated by respondents in most instances. The study revealed low demand for RI antigens among some of the respondents in identifying and addressing barriers to immunization coverage.

Routine immunization is the bedrock of immunization. Stakeholders need to strive hard towards creating demand in the context of RI services to improve uptake of RI antigens. The effort in addressing challenges faced by immunization needs to be sustained to prevent vaccine-preventable diseases from causing outbreaks in high-risk communities [19].

Overall, there was a positive attitude change towards OPV programs among noncompliant caregivers in 2016 compared with 2013 when the unmet needs intervention was introduced in Kaduna state. Belief is difficult to change over time, which indicates why Kaduna state still has some pockets of noncompliant households. However, there was an improvement in the level of OPV uptake in the state (2015–2016) compared with 2 years earlier (2013–2014).

Lessons learnt from the use of the unmet needs questionnaire points to the need to create demand for a routine immunization program for good coverage of other antigens in addition to OPV. High demand for RI antigens will curb the incidence of vaccine-preventable diseases from noncompliant communities and further sustain the effort gained in the fight against poliomyelitis. Integration of other health interventions such as a malaria program and improving the primary healthcare delivery system by provision of essential drugs at no charge alongside immunization services will also go a long way in reducing the burden of most childhood vaccine-preventable diseases in high-risk communities in Kaduna state.

Furthermore, noncompliant communities in the state have informed us of the need for a holistic approach. Policy makers need to collaborate with other units in the healthcare service delivery system and also with other sectors, especially areas that affect the general wellbeing of the populace that has a direct or indirect bearing on health determinants. Factors affecting health determinants are the provision of portable water, power supply, and good infrastructure. The majority of respondents in our study felt they had been deprived of such amenities by the government and craved for these services before they can allow vaccination of their children.

A major limitation of the study is a failure to use the appropriate format of the recommended unmet needs template for capturing the correct data by some of the interviewers at the initial stage which led to 504 datasets being discarded as a result of low quality. Also, it is possible that other important reasons why caregivers decline OPV for their children were not captured on the unmet needs template. Therefore, it is possible that some vital information could be missing for addressing noncompliance as a whole in some remaining noncompliant high-risk areas in Kaduna state.

## Conclusion

The unmet needs tool was effective in reducing noncompliant households by 79% in Kaduna state. The unmet needs tool should be applied continuously to guide immunization services in the state. The method can be adapted for use in other health programs facing similar challenges. Also, partnering with other donor-supported programs that provide health infrastructure or community development programs to very poor disadvantaged communities can alleviate the distress in such communities.

## Abbreviations

ERC: Expert Review Committee
FV: Field volunteer
GPEI: Global polio eradication initiative
LGA: Local Government Area
OPV: Oral polio vaccine
RI: Routine immunization
SIA: Supplemental immunization activity
WHO: World Health Organization
WPV: Wild poliovirus
